# Polyamines Disrupt the KaiABC Oscillator by Inducing Protein Denaturation

**DOI:** 10.3390/molecules24183351

**Published:** 2019-09-14

**Authors:** Jinkui Li, Lingya Zhang, Junwen Xiong, Xiyao Cheng, Yongqi Huang, Zhengding Su, Ming Yi, Sen Liu

**Affiliations:** 1 Key Laboratory of Fermentation Engineering (HBUT, Ministry of Education) and National “111” Center for Cellular Regulation and Molecular Pharmaceutics, Hubei University of Technology, Wuhan 430068, China; 2 Hubei Key Laboratory of Tumor Microenvironment and Immunotherapy, Medical College of China Three Gorges University, Yichang 443002, China; 3 Institute of Biomedical and Pharmaceutical Sciences, Hubei Key Laboratory of Industrial Microbiology, Hubei University of Technology, Wuhan 430068, China; 4 School of Mathematics and Physics, China University of Geosciences, Wuhan 430074, China

**Keywords:** polyamine, kai proteins, circadian clock, biological module, protein stability

## Abstract

Polyamines are positively charged small molecules ubiquitously existing in all living organisms, and they are considered as one kind of the most ancient cellular components. The most common polyamines are spermidine, spermine, and their precursor putrescine generated from ornithine. Polyamines play critical roles in cells by stabilizing chromatin structure, regulating DNA replication, modulating gene expression, etc., and they also affect the structure and function of proteins. A few studies have investigated the impact of polyamines on protein structure and function previously, but no reports have focused on a protein-based biological module with a dedicated function. In this report, we investigated the impact of polyamines (putrescine, spermidine, and spermine) on the cyanobacterial KaiABC circadian oscillator. Using an established in vitro reconstitution system, we noticed that polyamines could disrupt the robustness of the KaiABC oscillator by inducing the denaturation of the Kai proteins (KaiA, KaiB, and KaiC). Further experiments showed that the denaturation was likely due to the induced change of the thermal stability of the clock proteins. Our study revealed an intriguing role of polyamines as a component in complex cellular environments and would be of great importance for elucidating the biological function of polyamines in future.

## 1. Introduction

Polyamines are a kind of polycationic molecule which plays important roles in various cellular processes including stabilizing chromatin structure, regulating DNA replication, modulating gene expression, and interfering cell cycle [1]. Nonetheless, the biological role of polyamines needs further investigation [2]. Polyamines universally exist in nearly all living organisms including cyanobacteria and human cells, and the best-known polyamines are putrescine, spermidine, and spermine [3]. Recently, spermidine was proved to be useful in extending life spans in various animal models [4]. A few studies have shown that polyamines can induce changes in the structure and function of proteins [5,6,7,8], but how polyamines affect the biological function of protein-protein interaction modules has not been studied.

Circadian rhythms are the endogenous oscillations of biological reactions and behaviors in most organisms. On Earth, circadian rhythms have a period of around 24 h, and they play critical roles in regulating cellular functions and gaining evolutionary advantages [9]. Functionally, circadian rhythms should be robust and be able to tolerate environmental noises, such as temperature fluctuation, variation of cellular contents, and change of osmotic pressure [10]. Under the hood, circadian rhythms are controlled by circadian clocks [11]. A circadian clock is a molecular machine consisting of a set of macromolecules that can generate an oscillation with a ~ 24 h period and output the timing signal via downstream signal transduction. Logically, to generate robust circadian rhythms, a robust circadian clock is a necessity. Although recent studies have revealed the molecular mechanism of circadian clocks including both the transcriptional-translational feedback loop (TTFL) model and the post-translational oscillation (PTO) or non-TTFL model, how circadian clocks maintain their own robustness in complex cellular environments is not well studied thus far.

The circadian oscillator of cyanobacteria is an intensively studied model system for investigating the molecular mechanism of circadian clocks. Cyanobacteria are the simplest organism with confirmed circadian rhythms, and the core oscillator of cyanobacterial circadian clock consists of three clock proteins: KaiA, KaiB, and KaiC. The main oscillation of the KaiABC oscillator is the cycle of the phosphorylation/de-phosphorylation of KaiC. KaiC has both auto-kinase activity and auto-phosphatase activity [12]. KaiA binds to KaiC to stimulate the auto-phosphorylation of KaiC, whereas KaiB antagonizes KaiA’s function to initiate the de-phosphorylation of KaiC [13]. The most amazing aspect of the cyanobacterial circadian clock is that the circadian oscillator could be reconstituted in vitro with these purified clock proteins and ATP even without any DNA or RNA [14]. Previous studies showed that the in vitro KaiABC oscillator has the core characteristics of in vivo circadian clocks, i.e., free running, temperature compensation, and entrainment by environment cues [15]. Therefore, these studies proved that the in vitro KaiABC oscillator is a robust molecular system (or functional module) [16].

Cyanobacteria are divided into two groups based on their nitrogen metabolism categories: One is nitrogen-fixing and the other is non-nitrogen-fixing [3]. The polyamine contents are quite different between nitrogen-fixing and non-nitrogen-fixing cyanobacteria, and the major polyamines in non-nitrogen-fixing cyanobacteria are putrescine and spermidine [3]. *Synechococcus elongatus* PCC 7942 is a kind of non-nitrogen-fixing cyanobacteria, in which the spermidine concentration was determined to be ~20 nmol per 10^9^ cells, the putrescine concentration was ~6 × 10^−4^ µmol per 10^9^ cells, and spermine was not detectable [17]. In another analysis [18], *Synechococcus leopoliensis* had spermidine at 0.5 µmol per 1 g of wet cells (the individual cyanobacterial cell weight was ~4 × 10^−11^ g/cell [19]) and putrescine at 0.05 µmol per 1 g wet cells, and spermine was not detectable in the *Synechococcus* specie. Therefore, polyamines might be important players in cyanobacteria.

In this work, we set out to use the in vitro KaiABC oscillator to investigate the impact of polyamines on protein-protein interaction modules with dedicated biological functions. Our data showed that polyamines could disturb the in vitro KaiABC oscillator. We further uncovered that the disturbance was caused by the denaturation of the clock proteins induced by polyamines. At last, we showed that this role of polyamines is not universal for all proteins but likely dependent on proteins. Considering polyamines are indispensable and exist in nearly all known organisms with high cellular concentrations (especially in fast growing cells such as tumor cells), our work could be of great value in the future study on the biological function of polyamines.

## 2. Results

### 2.1. Polyamines can Disrupt the KaiABC Oscillator

From the data of cyanobacteria mentioned in the introduction section, an estimation of the putrescine amount in cyanobacterial cells would be around 0.02 µmol per 10^9^ cells. As determined previously [19], the cyanobacterial cells had an average cell size of 2 µm, then the cellular concentration of spermidine in cyanobacteria would be around 5 × 10^3^ µM. The cellular concentration of putrescine was around 20-fold less than spermidine [3], so the reference concentration of putrescine in cyanobacterial cells was estimated to be around 400 µM. These concentrations are comparable to the polyamine concentrations in normal human cells which are up to millimolar ranges [20]. However, the polyamine contents in human cells are quite different, and in specific cells such as tumor cells, the polyamine concentrations are even higher. For example, the concentration of polyamines could be up to 2 × 10^4^ µM in prostate cells [20]. Therefore, we set out to use up to 1 × 10^4^ µ M of polyamines for the analyses in this work.

**Figure 1:**
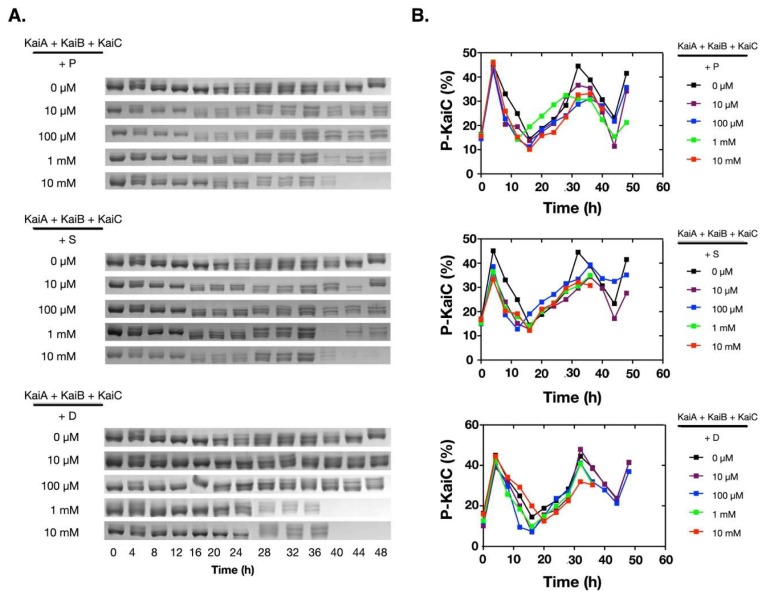
Polyamines disrupted the in vitro KaiABC oscillator at 1 mM and/or 10 mM. The purified Kai proteins (KaiA, KaiB, and KaiC) were mixed and incubated at 30 °C. The phosphorylation states of KaiC were analyzed by SDS-PAGE, and the percentage of the phosphorylated KaiC (P-KaiC) was determined with ImageJ (NIH). The upper bands were P-KaiC, and the lower bands were de-phosphorylated KaiC. The SDS-PAGE gels containing the KaiC bands were shown in (**A**), and the corresponding quantitative analyses were shown in (**B**). P: Putrescine; S: Spermine; D: Spermidine.

To assess how polyamines affect the oscillation of the in vitro KaiABC oscillator, we added different concentrations of polyamines into the reconstitution system of the Kai proteins from *Synechococcus elongatus* PCC 7942. Besides putrescine and spermidine, we also added spermine for comparison. As shown in Figure 1A, at low concentrations (10 µM and 100 µM), all three polyamines did not significantly affect the phosphorylation/de-phosphorylation oscillation of KaiC. When the concentrations were increased to 1.0 mM or 10 mM, no significant changes were detected on the oscillation of the KaiABC system in the first cycle (Figure 1B), but KaiC started to disappear after ~ 40 h of incubation. These data indicated that polyamines disrupted the robustness of the in vitro KaiABC oscillator.

### 2.2. Polyamines did not Directly Disturb the Functions of the Clock Proteins

**Figure 2:**
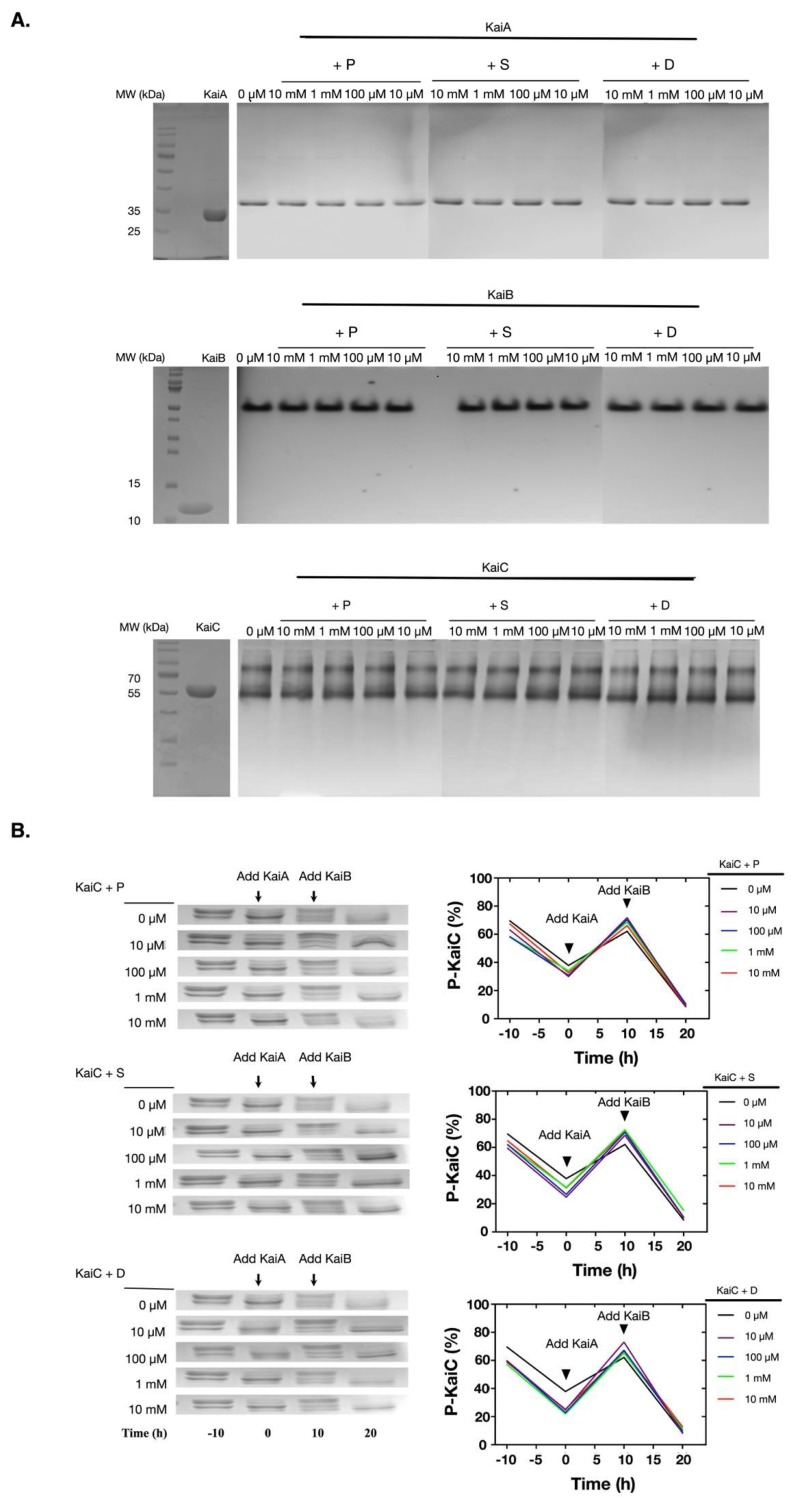
Polyamines did not directly interfere the functions of the Kai proteins. (**A**) The Kai proteins were incubated with polyamines for 4 h and then the oligomeric states of Kai proteins were analyzed with native PAGE gels. The protein names are noted above the top lines. Kai proteins did not show significant changes in their oligomeric states. The Kai proteins were highly pure as shown in the left SDS-PAGE gels with molecular markers. (**B**) The Kai proteins were incubated with polyamines for 4 h, and the activities of the Kai proteins were analyzed. When incubated alone at 30 °C, KaiC underwent de-phosphorylation. After the addition of KaiA, the phosphorylation of KaiC was stimulated. With the addition of KaiB, KaiC de-phosphorylated again. The upper bands were P-KaiC, and the bottom bands were de-phosphorylated KaiC. P: Putrescine; S: Spermine; D: Spermidine.

Since the previous experiment showed that polyamines could disturb the reconstituted KaiABC oscillator, we asked how polyamines affect the clock functions of the Kai proteins. We first asked if polyamines could directly disrupt the oligomerization of Kai proteins, since KaiA functions as dimers, KaiB as tetramers or dimers, and KaiC as hexamers [21]. As shown in Figure 2A, none of these polyamines disrupted the oligomerization states of the Kai proteins as determined by the native PAGE electrophoresis. We then asked if there were functional changes of Kai proteins under these conditions. As shown in Figure 2B, polyamines did not significantly cause changes in the functions of Kai proteins. No matter if polyamines were existing, KaiC underwent auto-dephosphorylation at 30 °C when being incubated alone, KaiA stimulated the phosphorylation of KaiC, and KaiB antagonized the function of KaiA.

### 2.3. Polyamines Induced Denaturation of the Clock Proteins

To test if the polyamine-induced protein denaturation was time dependent, we continued to check what happened to the solution system after long-time incubation. We incubated the Kai proteins alone or with 10 mM of polyamines for 72 h, before the samples were centrifuged at 12,000 rpm for 10 min, and the supernatants were analyzed with SDS-PAGE gels. As shown in Figure S1A, we noticed that Kai proteins became less or invisible in the supernatants, and when the precipitates were analyzed, Kai proteins were detected in many cases. This indicated that the proteins in these samples precipitated. Then we measured the changes of the pH values of the solutions using high accuracy pH test strips (resolution: 0.5 pH unit). Surprisingly, as shown in Figure S1B, the pH values of the mixtures of the Kai proteins and the polyamines dropped significantly, whereas the pH values were quite stable when the Kai proteins or the polyamines were incubated alone.

**Figure 3:**
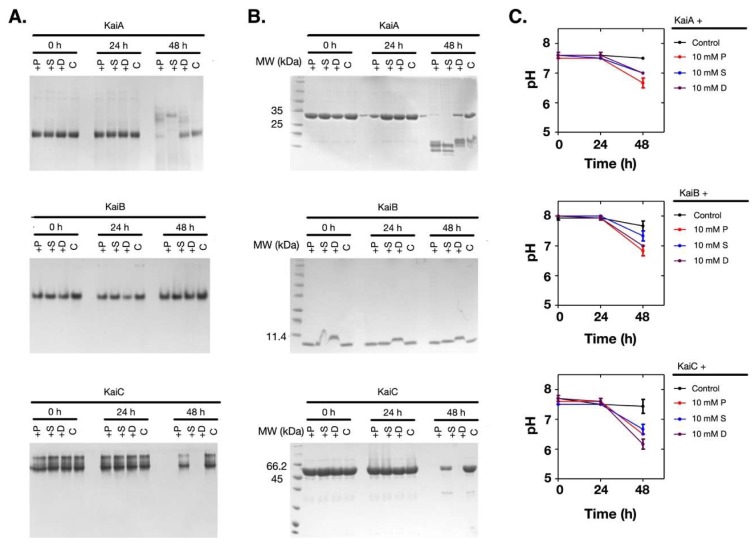
Polyamines caused the pH decrease of the solution and protein denature at 10 mM. The Kai proteins were incubated with 10 mM of polyamines. (**A**) The native gel analysis. (**B**) The SDS-PAGE analysis. (**C**) The changes in the pH values of the solutions. The protein names are noted above the top lines. P: Putrescine; S: Spermine; D: Spermidine; C: Control.

Supposing that the pH change of the solution was coupled with the denaturation of the proteins, we measured the time-dependent changes of the pH values and the proteins. As shown in Figure 3A–C, when the pH value of the solution dropped at 48 h (Figure 3C), both KaiA and KaiC had obvious changes in their oligomerization states and the soluble amounts (Figure 3A,B). However, KaiB was relatively stable. Therefore, these results showed that when the Kai proteins were incubated with polyamines at 30 °C, the pH changes and the denaturation of the Kai proteins were dependent on polyamines, but the protein denaturation was not caused by the pH change of the solution.

### 2.4. The Polyamine-Induced Denaturation is Protein-Dependent

**Figure 4:**
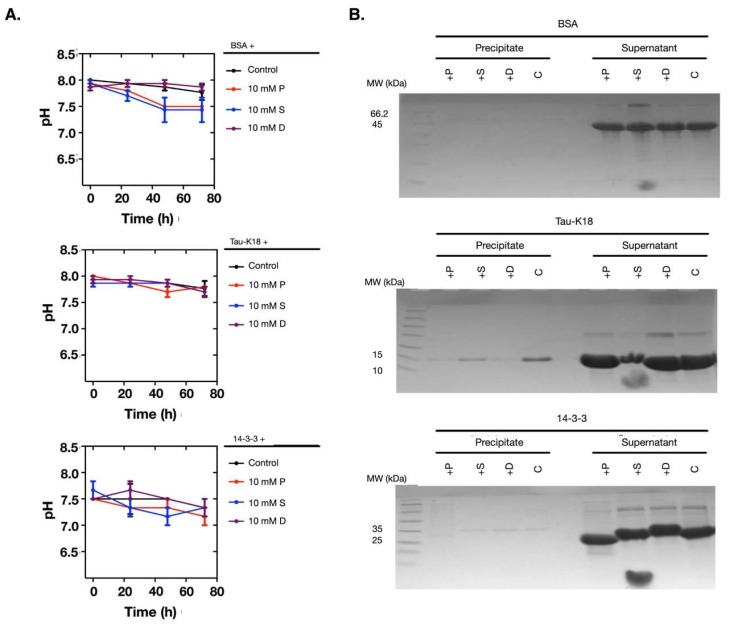
At 10 mM, polyamines did not cause pH changes and protein denaturation when incubated with BSA, Tau-K18, and 14-3-3. (**A**) The pH values of the solutions did not change when 10 mM of polyamines were incubated with BSA, Tau-K18, or 14-3-3. (**B**) The SDS-PAGE analyses showed that BSA, Tau-K18, and 14-3-3 were stable in the supernatants. The protein names are noted above the top lines. P: Putrescine; S: Spermine; D: Spermidine; C: Control.

To investigate if the pH change and the protein denaturation caused by polyamines were protein-dependent, we used more proteins for comparison. We chose bovine serum albumin (BSA), the human tau protein, and the 14-3-3 protein. Following the same procedures, we noticed that these proteins were more stable during the incubation and the pH values did not change (Figure 4A,B). Therefore, this result proved that the pH change and the protein denaturation caused by polyamines were protein-dependent.

### 2.5. Polyamines Induce the Decrease of the Thermal Stability of the Clock Proteins

**Figure 5:**
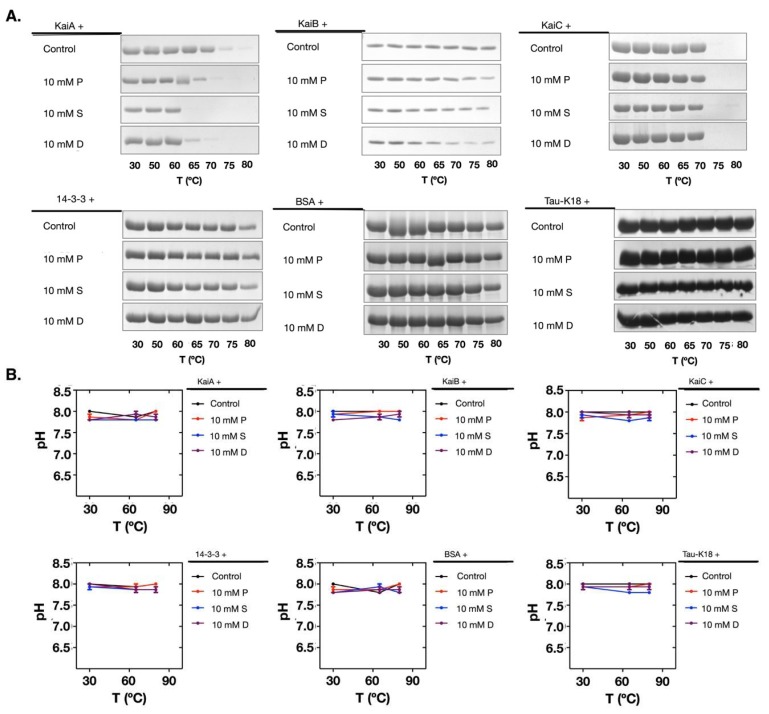
Polyamines induced the decrease of the thermal stability of Kai proteins. (**A**) The tested proteins showed differences in thermal stability when incubated with polyamines. (**B**) The pH values did not change during the thermal stability test for all proteins. The protein names are noted above the top lines. P: Putrescine; S: Spermine; D: Spermidine.

Polyamines are poly-cationic in the reconstitution system (pH 8.0) according to the pKa values of the amino groups (putrescine: 10.80 and 9.35; spermidine: 10.9, 8.4, and 9.9; and spermine: 10.9, 8.4, 7.9, and 10.1) [22]. Therefore, if a protein has more acidic (negatively charged) residues, it could form complexes with polyamines more easily. However, the surface electrostatic potentials of the tested proteins could not explain the experimental data (Figure S2). Similarly, the charged residues and the isoelectric points (pIs) of these proteins were not good indicators as well (Tables S1 and S2). Then we tested if polyamines destabilized the Kai proteins. As shown in Figure 5A, polyamines decreased the thermal stability of KaiA and KaiC, whereas KaiB was more stable. In previous tests (Figure 3A,B), we also noticed that during the incubation of the Kai proteins with polyamines, KaiB showed relatively higher stability than KaiA and KaiC. Supporting the hypothesis that pH change is not a direct cause of the denature of Kai proteins, the pH values were stable during the thermal stability test (Figure 5B). Therefore, the denaturation of the Kai proteins was likely caused by the decreased thermal stability induced by polyamines.

## 3. Discussion

Polyamines are indispensable for virtually all known organisms, and in mammalian cells and tissues, polyamine concentrations are up to millimolar ranges [20]. In human, the polyamine concentration can be up to 5 mM in nucleus and even as high as 20 mM in prostate cells [20]. The concentration of polyamines is stringently controlled by the sophisticated polyamine network. However, the network is deregulated in tumor cells and the polyamine concentrations in tumor cells are much higher than normal cells [20]. On the other side, the polyamine concentration decreases during the aging process. Recent studies have shown that dietary spermidine supplementation could improve cardiovascular function, ameliorate memory impairment, and delay aging process [23,24,25]. Therefore, the biological function of polyamines has attracted increased attention in recent years [1,24,26].

Previously, a few reports paid attention to the impact of polyamines on protein structure and function [5,6,7,8]. In this work, we went deeper to investigate how polyamines affect the function of a protein-based biological module. To be simple, we chose the KaiABC oscillator of the cyanobacterial circadian clock since it could be reconstituted in vitro with the purified proteins. More importantly, the reconstituted system is fully functional as a robust functional module. With this system, our study revealed that polyamines disrupted the robustness of this system by inducing the denaturation of the Kai proteins. Further tests suggested that the denaturation was due to the decreased thermal stability of the clock proteins induced by polyamines. However, this effect of the polyamines was protein dependent.

Structurally, these three polyamines are different in chain length and charge states, so their functions are different. For example, spermidine, but not putrescine and spermine, can significantly extend the life span of animals [27]. Regarding affecting the structure and function of proteins, it was also noticed there exist some differences between these polyamines [7]. It was also found that polyamines might have specific binding sites on proteins [6,28]. In our work, although there were some differences in several samples between different polyamines, the overall differences were not significant. We will continue to study if there exist specific interactions between polyamines and Kai proteins.

What structural characteristics caused the difference between KaiA/KaiC and KaiB in this work? It has been known that KaiA and KaiC are mainly homo-oligomers and the monomeric forms are not detectable, whereas KaiB has relatively stable monomeric form and has the monomer-dimer-tetramer equilibrium [29]. Similarly, the monomeric forms of BSA and Tau-K18 are stable, and 14-3-3 has detectable monomeric form forming a dynamic monomer-dimer equilibrium. Therefore, the role of polyamines might be correlated with the intrinsic structural stability of the target protein.

One interesting question stimulated by our work is how polyamines affect the cellular environment and protein aggregation in living cells. As mentioned above, cellular polyamines could be up to 20 mM. Then how those polyamines affect the structure and function of different cellular proteins would be an intriguing topic to study. For instance, is the formation of amyloid-beta fibrils in brain cells caused or stimulated by polyamines [30]. In tumor cells, the cellular pH values are lower than normal cells [31], so do these changes resonate with the elevated level of polyamines? Additionally, what caused pH drops during the incubation of polyamines and Kai proteins needs further investigations.

In conclusion, our work disclosed an interesting role of polyamines in regulating protein structure and function. And this finding would be of great value to the elucidation of the biological role of polyamines.

## 4. Materials and Methods

### 4.1. Reagents

The polyamines (putrescine, spermidine, and spermine) used in this work were purchased from Sigma-Aldrich (St. Louis, MO, USA). The other chemical reagents for protein purification and buffer preparation were purchased from Sinopharm (China) and Shanghai Bioengineering Co., Ltd. (Shanghai, China). BSA was purchased from Shanghai Bioengineering Co., Ltd. (Shanghai, China).

### 4.2. Protein Expression and Purification

The pGEX-6p-1 plasmids containing the coding sequence of KaiA, KaiB, and KaiC proteins from *Synechococcus elongatus* PCC 7942 were kindly provided by Dr. Carl Johnson (Vanderbilt University, Nashville, TN, USA), and the expression and purification procedure was similar as reported in our previous work [32,33]. Briefly, the Kai proteins were expressed as GST-tagged proteins in *E. coli* and purified with GSH resins. The GST tag was removed by the PreScission protease, and the tag-free proteins were purified with size-exclusion chromatography.

The coding sequences of human-derived Tau-K18 and 14-3-3 proteins were inserted in the pRSV plasmid for expressing the corresponding target proteins with a 6xHis tag at the N-terminus. The plasmids were transformed into BL21(DE3), and monoclones were picked and cultured in LB (Luria-Bertani) liquid medium. The expression of the proteins was induced with 0.2 mM of Isopropyl β-d-1-thiogalactopyranoside (IPTG) at 30 °C for 8 h. The target proteins were purified with a Ni-NTA affinity column, followed by an ion exchange column. For Tau-K18, before the Ni-NTA purification, the sample was incubated at 95 °C for one hours and centrifuged to pre-purify the protein. All plasmids were verified by DNA sequencing. The detailed sequences and cloning information of the proteins are included in Table S1.

### 4.3. Sample Preparation

The buffer used for protein incubation was 50 mM Tris-HCl (pH 8.0), 150 mM NaCl, 5 mM ATP, 5 mM MgCl_2_, and 0.01% Tween-20. The polyamines (putrescine, spermidine, and spermine) were first dissolved to 100 mM in this buffer before being supplemented to indicated samples. All samples were incubated at 30 °C in a PCR machine with heated lid, and aliquots were collected at indicated time. The in vitro KaiABC oscillator was reconstituted as described in our previous paper [32]. The concentration ratio of Kai proteins (KaiA:KaiB:KaiC) was 1:1:2 (*m*/*v*) when two or three of them were mixed. BSA, Tau-K18, and 14-3-3 proteins were processed similarly. In all samples, the total protein concentration was adjusted to 1.0 mg/mL. For the thermal stability experiment, the samples were incubated at the indicated temperatures for 7–40 min before the samples were collected for analysis.

### 4.4. Polyacrylamide Gel Electrophoresis of the Protein Samples

The collected samples at different time points were stored at −20 °C before being analyzed with 12% SDS-PAGE gels or 6% native PAGE gels. For samples with precipitates, the samples were centrifuged at 12,000 rpm for 5 min before the supernatants were aliquoted out for analysis. The precipitates were dissolved with 8 M of urea and then subjected to SDS-PAGE analysis.

### 4.5. Analysis of the Phosphorylation Level of KaiC

The SDS-PAGE gels for determining the phosphorylation level of KaiC were analyzed in ImageJ (NIH, Bethesda, MD, USA) as described in our previously published protocol [34].

## Supplementary Materials

The following are available online at https://www.mdpi.com/1420-3049/24/18/3351/s1, Figure S1: Polyamines caused the drop of solutions’ pH values and protein denature after long-time incubation with Kai proteins, Figure S2: The electrostatic potentials of the protein surfaces were calculated with APBS (v2.1) in Pymol with the default settings, Table S1: The sequences and cloning information of the proteins tested in this study, Table S2: The sequence compositions of the tested proteins could not explain the role of polyamines.
