# Supplementary material for: Polyamines Disrupt the KaiABC Oscillator by Inducing Protein Denaturation
Source: Molecules. 2019 Sep 14;24(18):3351. doi: 10.3390/molecules24183351 (PMC6767301; doi:10.3390/molecules24183351)
Supplement: Supplementary file 1 [file molecules-24-03351-s001.pdf]

Supplementary materials

Polyamines disrupt the KaiABC oscillator by inducing protein denaturation

Jinkui Li[1,2], Lingya Zhang[2], Junwen Xiong[1,3], Xiyao Cheng[1,3], Yongqi Huang[1,3], Zhengding Su[1,3], Ming Yi[4,*], Sen Liu[1,2,3,*]

[1] Key Laboratory of Fermentation Engineering (HBUT, Ministry of Education) and National "111" Center for Cellular Regulation and Molecular Pharmaceutics, Hubei University of Technology, Wuhan 430068, China

[2] Hubei Key Laboratory of Tumor Microenvironment and Immunotherapy, Medical College of China Three Gorges University, Yichang 443002, China

[3] Institute of Biomedical and Pharmaceutical Sciences, Hubei Key Laboratory of Industrial Microbiology, Hubei University of Technology, Wuhan, 430068, China

[4] School of Mathematics and Physics, China University of Geosciences, Wuhan 430074,China

* Correspondence: senliu.ctgu@gmail.com (S.L.); mingyi@cug.edu.cn (M.Y.)

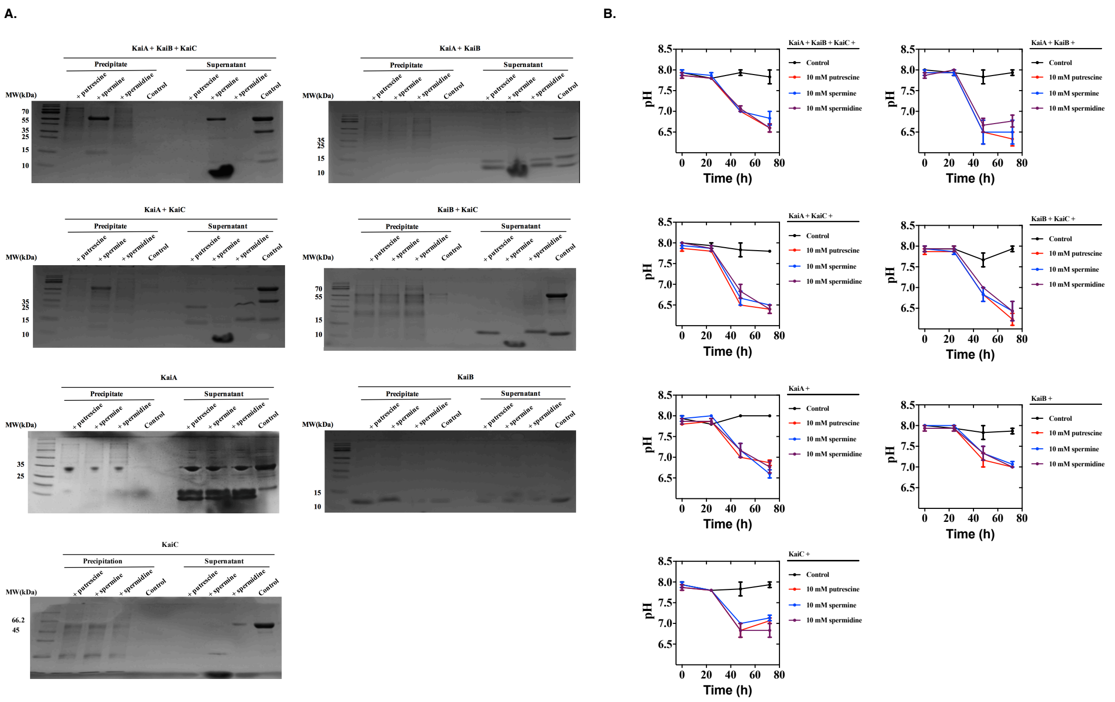

Figure S1. Polyamines caused the drop of solutions' pH values and protein denature after long-time incubation with Kai proteins. The samples without extra polyamines were controls. The total protein amounts of each lane were same. (A) The SDS-PAGE gels showed that the Kai proteins became less or invisible in the supernatants after being incubated for 72 hours with 10 mM of polyamines. Kai proteins became less or invisible in the supernatants. The additional protein bands at ~ 15 kDa were likely from the degradation of KaiA. (B) The pH values of the mixtures of Kai proteins and 10 mM polyamines dropped significantly after long-time incubation. The protein names are noted above the top lines.

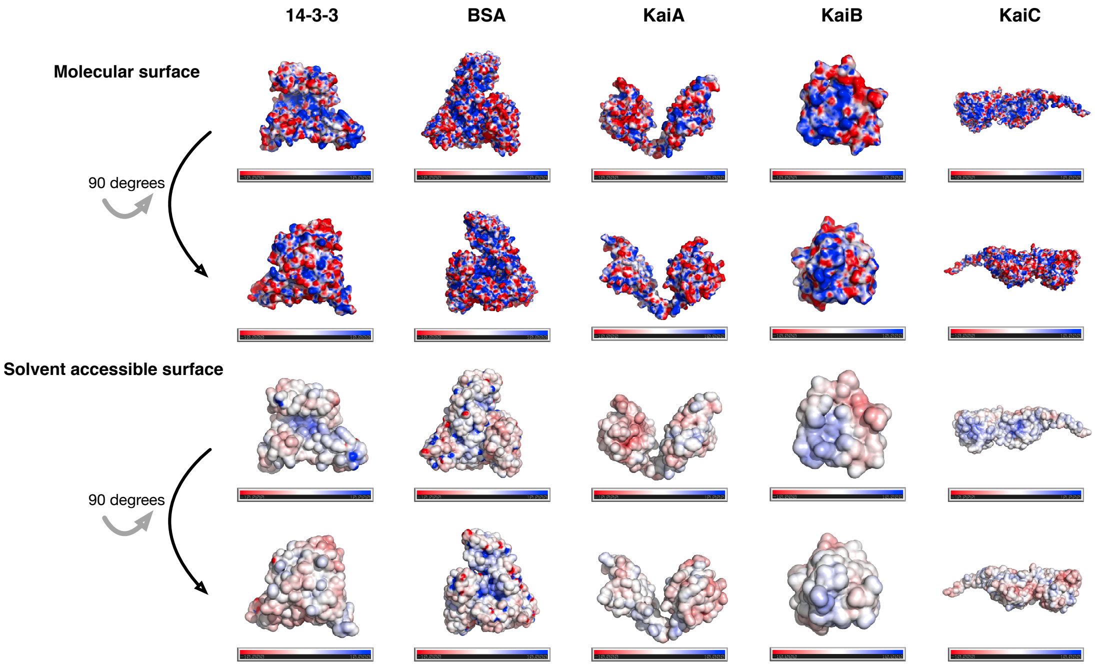

Figure S2. The electrostatic potentials of the protein surfaces were calculated with APBS (v2.1) in Pymol with the default settings. For each Kai protein, only a monomer in the homo-oligomers was shown. The Tau-K18 protein was not shown because its apo structure was not available. The PDB IDs were: 14-3-3, 3RDH; BSA, 3V03; KaiA, 5C5E; KaiB, 1R5P; KaiC, 2GBL.

Table S1. The sequences and cloning information of the proteins tested in this study.

Protein	Source	DNA coding sequence	Protein sequence	Restriction sites	Plasmid
KaiA	*Synechococcus sp.* PCC 7942	ctctcgcaaattgcaatctgcatttgggtggaatcgacggcaattttgcaggattgccagcgggcgctgtcggccgatcgctatcaactccaagtctgtgagtctggcgaaatgctcttggagtatgcccaaacccatcgtgaccaaatcgactgcctgattttagtggcagccaatcccagcttcagggcagttgttcagcagctctgctttgagggagtggtggtaccagcgattgtcgtaggcgatcgcgacagtgaggatcccgatgaaccagccaaagaacagctctatcacagcgctgaactgcacctcggtatccatcagctcgagcaattgccctaccaagttgatgctgcactggctgaatttctgcgcttagccccggtcgagaccatggccgaccacatcatgctgatgggggccaaccacgatcccgagctatcgagccagcagcgggacctcgctcagcgactacaagagcgcctaggctatctcggggtctactacaagcgtgatcccgatcgctttctgcgcaacctacccgcctacgaaagccaaaagctgcaccaagcgatgcagactagctatcgtgaaatcgttttgagctatttttcgccgaatagcaacctcaaccagagcattgacaacttcgtcaacatggctttctttgccgatgttccagtcaccaaagtggtagaaattcacatggagctgatggacgagtttgccaagaagctccgcgtagagggacgttcagaggacattttgctggattatcggctgactttaattgatgtaattgcacatctttgtgagatgtatcgacggtctatcccacgagaaacctga	GPLGSLSQIAICIWVESTAILQDCQRALSADRYQLQVCESGEMLLEYAQTHRDQIDCLILVAANPSFRAVVQQLCFEGVVVPAIVVGDRDSEDPDEPAKEQLYHSAELHLGIHQLEQLPYQVDAALAEFLRLAPVETMADHIMLMGANHDPELSSQQRDLAQRLQERLGYLGVYYKRDPDRFLRNLPAYESQKLHQAMQTSYREIVLSYFSPNSNLNQSIDNFVNMAFFADVPVTKVVEIHMELMDEFAKKLRVEGRSEDILLDYRLTLIDVIAHLCEMYRRSIPRET	*BamH* I<br>*Not* I	pGEX-6P-1
KaiB	*Synechococcus sp.* PCC 7942	agccctcgtaaaacctacattctcaagctctacgtcgccggcaatactccaaactcagtccgtgccctcaaaacgctcaagaacattctcgaagttgaatttcaaggtgtttatgctctaaaggtgatcgatgttctcaaaaatcctcagttggcagaagaggataaaatcctagcgacgccaaccctcgccaaggttctaccactgcctgtccgacggattattggtgatttatccgaccgtgagaaagttttgattggccttgatttactctacggcgaacttcaagattccgacgacttctaa	GPLGSSPRKTYILKLYVAGNTPNSVRALKTLKNILEVEFQGVYALKVIDVLKNPQLAEEDKILATPTLAKVLPLPVRRIIGDLSDREKVLIGLDLLYGELQDSDDF	*BamH* I<br>*Not* I	pGEX-6P-1

KaiC	*Synechococcus sp.* PCC 7942	ggatccacttccgctgagatgactagccctaataataattctgagcaccaagccatcgctaag<br>atgcgcacgatgattgaaggctttgatgatattagtcatggcggtcttccaatcgggcgatcga<br>ccctcgttagtggtacttcaggaaccggcaagacccttttttctattcaatttctctataacggtatt<br>atcgagtttgatgagcctggggttttcgttactttcgaagaaaccccgcaagatatcattaaaaa<br>cgcccgtagttttggctgggatttagccaagctggtcgatgagggcaaactatttattcttgatg<br>cttcacccgatccagaaggtcaagaggttgttggcggcttcgatctctctgctctgattgagcg<br>gattaattatgcaattcaaaagtatcgagcgcggcgggtttcaattgactcggtcacgtccgttt<br>tccagcaatatgatgcctcttctgtggttcgccgcgaactctttcggttggtagctcgcctaaaa<br>caaattggggcaactacggtcatgaccaccgagcgtatcgaggaatatggcccgatcgctc<br>gttacggtgttgaggaatttgtctccgataacgtcgtgattctccgcaacgttttggaaggggga<br>gcgccgtcgccgcaccctcgaaatcctcaagctacgtggcaccagccacatgaaaggggga<br>atatccgttcacgattacggatcatggcatcaatatcttcccgctcggggcaatgcgccttacg<br>cagcgatcgtcgaacgtgcgtgtttcatctggtgtcgtccgactcgatgaaatgtgtggtggg<br>ggcttctttaaggactcaatcattctggcaactggcgctacaggcactggtaaaactctgttagt<br>tagccgtttcgttgagaatgcttgtgctaacaaagagcgggcgattctgttcgcttatgaagagt<br>cacgagctcagctgctccgcaatgcctattcatggggaatggactttgaggagatggagcgc<br>caaaacctcctcaaaattgtttgcgcctatcctgaatctgcaggtcttgaagaccatttgcagatt<br>attaagtcggagatcaatgactttaagccagctcgtattgcaatcgactccctctctgctttggc<br>gcgggggcgttagcaacaatgccttccgccaatttgtaattggtgtcactggctacgcgaaaca<br>agaagaaatcacgggactattcacaaataccagtgatcaatttatgggagcgcattcgattact<br>gactcccatatctcaacaattacggatacgattatcttgctccaatacgtcgagattcgtggcga<br>aatgtcccgcgccattaacgtcttcaagatgcgcggatcttggcatgacaaagcaatccgcga<br>attcatgatcagcgacaaagggccggacatcaaggattctttccggaactttgagcggattatt<br>tcaggttcgccaacacggattaccgtcgatgagaaaagcgaactctcgcgaattgtgcgcgg<br>cgttcaagaaaaagggccggagagctagcca	GPLGSGSTSAEMTSPNNNSEHQAI<br>AKMRTMIEGFDDISHGGLPIGRST<br>LVSGTSGTGKTLFSIQFLYNGIIEF<br>DEPGVFVTFEETPQDIIKNARSFG<br>WDLAKLVDEGKLFILDASPDPEG<br>QEVVGGFDLSALIERINYAIQKYR<br>ARRVSIDSVTSVFQQYDASSVVR<br>RELFRLVARLKQIGATTVMTTERI<br>EEYGPIARYGVEEFVSDNVVILRN<br>VLEGERRRRTLEILKLRGTSHMK<br>GEYPFTITDHGINIFPLGAMRLTQ<br>RSSNVRVSSGVVRLDEMCGGGFF<br>KDSIILATGATGTGKTLLVSRFVE<br>NACANKERAILFAYEESRAQLLR<br>NAYSWGMDFEEMERQNLLKIVC<br>AYPESAGLEDHLQIIKSEINDFKP<br>ARIAIDSLSALARGVSNNAFRQFV<br>IGVTGYAKQEEITGLFTNTSDQF<br>MGAHSITDSHISTITDTIILLQYVEI<br>RGEMSRAINVFKMRGSWHDKAI<br>REFMISDKGPDIKDSFRNFERIISG<br>SPTRITVDEKSELSRIVRGVQEKG<br>PES	*BamH* I<br>*Not* I	pGEX-6P-1

Tau-K18	Homo sapiens	atgggcagcagccatcaccatcatcaccacagccaggatctggaaaacctgtattttcaggg<br>atcccaaaccgcgccggtgccgatgccggacctgaaaaacgttaagagcaaaatcggtagc<br>accgagaacctgaaacaccagccgggtggcggtaaagtgcaaatcattaacaagaaactgg<br>acctgagcaacgttcagagcaagtgcggcagcaaagataacattaaacatgtgccgggcgg<br>tggcagcgtgcaaattgtttacaaaccggtggacctgagcaaagttaccagcaagtgcggta<br>gcctgggcaacatccaccacaagccgggtggcggtcaggttgaggtgaagagcgaaaaac<br>tggacttcaaagatcgtgtgcaaagcaagatcggtagcctggataacattacccacgttccgg<br>gcggtggcaacaagaaaattgaataa	MGSSHHHHHHSQDLENLYFQGS<br>QTAPVPMPDLKNVKSKIGSTENL<br>KHQPGGGKVQIINKKLDLSNVQS<br>KCGSKDNIKHVPGGGSVQIVYKP<br>VDLSKVTSK<br>CGSLGNIHHKPGGGQVEVKS<br>EKLDFKDRVQ SKIGSLDNIT<br>HVPGGGNKKIE	*BamH* I<br>*EcoR*I	pET-28a
14-3-3	Homo sapiens	atgggcagcagccatcaccatcatcaccacagccaggatctggaaaacctgtattttcaggg<br>atccatggacaagaacgagctggtgcagaaggcgaaactggcggagcaagcggaacgtta<br>cgacgatatggcggcgtgcatgaagagcgtgaccgagcagggtgcggaactgagcaacg<br>aggaacgtaacctgctgagcgttgcgtataaaaacgtggttggtgcgcgtcgtagcagctgg<br>cgtgtggttagcagcatcgaacaaaagaccgagggcgcggaaaagaaacagcaaatggc<br>gcgtgagtaccgtgaaaaaatcgagaccgaactgcgtgacatttgcaacgatgtgctgagcc<br>tgctggagaagttcctgatcccgaacgcgagccaggcggaaagcaaagtgttctacctgaa<br>gatgaagggtgactactatcgttatctggcggaagtggcggcgggtgacgataagaaaggc<br>attgttgatcagagccagcaagcgtaccaagaggcgttcgaaatcagcaagaaagaaatgc<br>aaccgacccacccgattcgtctgggtctggcgctgaacttcagcgtgttctactatgagattct<br>gaacagcccggaaaaggcgtgcagcctggcgaaaaccgcgtttgacgaggcgatcgcgg<br>aactggataccctgagcgaggaaagctataaagacagcaccctgattatgcagctgctgcgt<br>gataacctgaccctgtggaccagcgacacccaaggtgatgaggcggaagcgggtgagggt<br>ggcgaaaactaa	MGSSHHHHHHSQDLENLYFQGS<br>MDKNELVQKAKLAEQAERYDD<br>MAACMKSVTEQGAELSNEERNL<br>LSVAYKNVVGARRSSWRVVSSIE<br>QKTEGAEKKQQMAREYREKIETE<br>LRDICNDVLSLLEKFLIPNASQAE<br>SKVFYLKMKGDYYRYLAEVAAG<br>DDKKGIVDQSQQAYQEAFEISKK<br>EMQPTHPIRLGLALNFSVFYYEIL<br>NSPEKACSLAKTAFDEAIAELDTL<br>SEESYKDSTLIMQLLRDNLTLWT<br>SDTQGDEAEAGEGGEN	*BamH* I<br>*EcoR*I	pET-28a

BSA	Bovine	Not applicable (Commercial product)	DTHKSEIAHRFKDLGEEHFKGLV<br>LIAFSQYLQQCPFDEHVKLVNEL<br>TEFAKTCVADESHAGCEKSLHTL<br>FGDELCKVASLRETYGDMADCC<br>EKQEPERNECFLSHKDDSPDLPK<br>LKPDPNTLCDEFKADEKKFWGK<br>YLYEIARRHPYFYAPELLYYANK<br>YNGVFQECCQAEDKGACLLPKIE<br>TMREKVLTSSARQRLRCASIQKF<br>GERALKAWSVARLSQKFPKAEF<br>VEVTKLVTDLTKVHKECCHGDL<br>LECADDRADLAKYICDNQDTISS<br>KLKECCDKPLLEKSHCIAEVEKD<br>AIPENLPPLTADFAEDKDVCKNY<br>QEAKDAFLGSFLYEYSRRHPEYA<br>VSVLLRLAKEYEATLEECCAKDD<br>PHACYSTVFDKLKHLVDEPQNLI<br>KQNCDQFEKLGEYGFQNALIVRY<br>TRKVPQVSTPTLVEVSRSLGKVG<br>TRCCTKPESERMPCTEDYLSLILN<br>RLCVLHEKTPVSEKVTKCCTESL<br>VNRRPCFSALTPDETYVPKAFDE<br>KLFTFHADICTLPDTEKQIKKQTA<br>LVELLKHKPKATEEQLKTVMENF<br>VAFVDKCCAADDKEACFAVEGP<br>KLVVSTQTALA	Not applicable	Not applicable

Table S2. The sequence compositions of the tested proteins could not explain the role of polyamines. (A) The sequence properties of the proteins studied in this work were compared. The parameters were calculated with ProtParam (http://web.expasy.org/protparam/). The sequences of the expressed proteins were used except BSA (Table S2). Glu and Asp were considered as negatively charged residues, whereas Arg and Lys were positively charged.

Name	Molecular weight (Da)	Theoretical pI	Positively charged residue ratio	Negatively charged residue ratio
KaiA	32912.52	4.83	8.68%	14.93%
KaiB	11716.69	6.47	13.21%	13.21%
KaiC	58427.31	5.81	11.81%	13.14%
BSA	66462.98	5.60	14.06%	16.98%
Tau-K18	16250.42	9.52	13.91%	8.61%
14-3-3	30312.80	4.93	11.61%	17.60%
